# The contractual vacuum: AI procurement and the erosion of good administration

**DOI:** 10.3389/frai.2026.1916534

**Published:** 2026-09-23

**Authors:** Mohammed Abdulbari

**Affiliations:** 1 Faculty of Law, King Abdulaziz University, Jeddah, Saudi Arabia; 2 Department of Public Law, King Abdulaziz University, Jeddah, Saudi Arabia

**Keywords:** administrative law, computational legal design, continuous legality, correction cost model (CCM), digital constitutionalism, evidentiary sovereignty, public procurement, three-layer accountability framework

## Abstract

The integration of artificial intelligence as a service (AIaaS) within public administration fundamentally relocates the state’s evidentiary and inferential capacities into proprietary software infrastructures. When public bodies deploy these third-party systems, they introduce an acute systemic risk: a structural “contractual vacuum” emerges wherein public authorities lack the practical data telemetry required to reconstruct, explain, or correct algorithmic administrative actions. This article evaluates the doctrinal limitations of contemporary European and common law public procurement frameworks, demonstrating that conventional commercial procurement defaults inherently thin down the non-delegable duty of good administration. To bridge the structural divide between administrative law and systems engineering, this study introduces the methodology of computational legal design through a nested Three-Layer Accountability Framework. This framework is defined as a conceptual technical architecture that translates core public law norms into automated engineering constraints. It is normatively proposed as a model code-level continuous compliance pipeline that utilises transitive audit scripts, legal sandboxes, and a stylised programmatic correction cost model (CCM) formula to demonstrate how public law remedies can be calculated through automated system telemetry.

## 1 Introduction

Public power must remain intelligible to officials, affected persons, and reviewing institutions. AI-as-a-Service (AIaaS) procurement for welfare triage, fraud detection, migration screening, taxation, policing, or prioritisation unsettles that premise because the evidentiary foundations of public power may move into proprietary infrastructures beyond full public control. The issue is not merely technical opacity, but the weakening of the legal architecture that makes power reasoned, reviewable, and corrigible (Harlow and Rawlings, 2022; Craig, 2021).

Public procurement increasingly concerns cloud-hosted systems, composable agent architectures, supplier-controlled retraining, subcontracted data chains, and update pipelines capable of altering the practical application of legal standards (Cobbe et al., 2023). In this machine-to-machine (M2M) and AI Agent Economy, administrative cognition may be distributed across private actors. What the state acquires is therefore not just software, but access to a continuously maintained decisional environment through which administrative competence is partly externalised (OECD, 2015; Office for Artificial Intelligence, 2020; Veale and Brass, 2019).

The central gap is contractual. Administrative law still requires reasons, records, fairness, equality, and review, but those duties become hollow if the authority has not secured the evidence, controls, and intervention rights needed after outsourcing. Whilst conventional accountability-gap literature focuses primarily on *post hoc* judicial review or ethical design principles (Cobbe et al., 2021; Citron, 2008; Pasquale, 2015), this article targets the procurement contract itself as a structural control point (Yeung, 2018). It demonstrates that standard commercial defaults actively facilitate an unaddressed contractual vacuum that conventional public law remedies fail to reach. Through the Holmesian strategic-actor lens, every silence in audit rights, update governance, or secrecy language invites strategic opacity (Holmes, 1897; Citron, 2008; Public Law Project, 2024). In a composable M2M economy, audit rights must be transitive so that the lead vendor guarantees inspection rights down the logic-chain to any sub-agents or third-party models materially contributing to the final administrative output (United Nations, 2011; Public Law Project, 2024; OECD, 2024; Pasquale, 2015, p. 6; Citron, 2008).

The thesis is that AIaaS procurement should be analysed as outsourcing administrative capacity. That outsourcing does not dilute the non-delegable core of public law; rather, this shift supports the argument that authorities should negotiate contractual rights for reason-giving, auditability, and remedial capacity to ensure public power remains reviewable. Where the state disables its own access to evidence by contract, it fractures the social contract of administration (Harlow and Rawlings, 2022; Hildebrandt, 2020). The analysis is both descriptive and normative, demonstrating how continuously updated, vendor-controlled AI systems generate an acute legality gap that orthodox administrative law struggles to repair *post hoc*. To address this vulnerability, this study argues for a non-derogable contractual core—encompassing reason-giving, non-discrimination, transparency, meaningful human oversight, traceability, and access to remedy—implemented through *ex ante* procedural validation, functional liability, and restitutive remedies (OECD, 2025a; Cabinet Office, 2026).

The proposed Three-Layer Accountability Framework serves as a normative design blueprint, testing how administrative justice principles can be mapped onto software engineering pipelines to simulate algorithmic accountability. Layer 1 establishes *ex ante* procedural validation to test algorithmic rationality prior to deployment. Layer 2 defines functional operational liabilities to secure continuous legality during active use. Layer 3 mandates programmatic restitutive remedies to repair administrative failures. The subsequent analysis is structured around an integrated methodology combining transparent corpus coding, jurisdiction-specific legal analysis, and technical feasibility hedging. By moderating categorical assertions into normative design blueprints, the following sections evaluate standardised public procurement templates against precise administrative law benchmarks, translating this nested model into an actionable compliance matrix supported by structured reference tables, verifiable arithmetic formulas, and a technical architecture schema (Pasquale, 2015; Yeung, 2018).

## 2 Methodological approach: computational legal design

Unlike conventional doctrinal legal analysis focusing exclusively on judicial interpretation, this study applies a Computational Legal Design methodology. It operationalises a qualitative content analysis of contractual frameworks, translating public-law norms into functional, code-level technical architecture constraints at the point of system origin. This approach models how procurement clauses function as technical governance specifications. It explores their theoretical translation into logging requirements, metadata schemas, and automated regression thresholds across seven control dimensions: explainability, non-discrimination, traceability, update governance, human oversight, remedial access, and exit continuity (Surden, 2014; Hildebrandt, 2020; ECMA International, 2017). By evaluating these instruments textually, the study maps how public law values can be programmatically embedded within software engineering pipelines.

To operationalise these dimensions, this study develops an original normative blueprint rather than describing historical commercial procurement defaults. These proposed instruments—detailed in subsequent sections—provide a pro-competitive technical architecture designed to test how public law obligations translate directly into software engineering specifications.

This article addresses a single institutional question: how must public law be translated into contract when administrative capacity is outsourced through AIaaS? The constitutionalist lens treats AIaaS contracting not as ordinary commercial deal-making, but as the externalisation of capacities through which the state hears, classifies, reasons, and acts. In a Dworkinian sense, the legal order is read as an integrated structure of principle (Dworkin, 1977), balanced against Holmes’s strategic-actor account of legal realism (Holmes, 1897). Where public power operates through private technical architecture, public law principles dictate that the contract must keep that power supervisable (Craig, 2021; Harlow and Rawlings, 2022; United Nations, 2011).

Consequently, model clauses and official frameworks serve as legal-institutional data showing how public authorities operationalise duties at purchase. The principal comparators are the European Commission’s model contractual clauses, the UK Guidelines for AI Procurement, and the OECD Recommendation on Public Procurement (European Commission, 2025b; Office for Artificial Intelligence, 2020; OECD, 2025a). These materials triangulate rights-based, principles-based, and transnational governance approaches to preserve the constitutional minimum of good administration. The doctrinal mechanisms for restricting privatisation differ sharply by territory. In the US, this is governed by the private non-delegation doctrine; UK and Commonwealth courts rely on the rule against contractually fettering statutory powers. Rather than blending these distinct frameworks, this study treats them as parallel doctrinal pathways reaching a shared normative conclusion.

To ensure a reproducible analysis, document retrieval was conducted via a systematic search of official central government procurement repositories, including the EU Public Buyers Community portal, the UK Government Digital Service and Cabinet Office repositories (Government Digital Service, 2026), the US General Services Administration portal, and the OECD AI Policy Observatory hub. Search strings combined public procurement terms (“procurement guidelines,” “model clauses”) with algorithmic identifiers (“artificial intelligence,” “AIaaS,” “machine learning”). The corpus inclusion boundary was restricted to English-language texts published or updated between January 2020 and June 2026 to capture regulatory shifts.

Consequently, this deductive comparative framework evaluates the definitive texts issued by central authorities in the US (US General Services Administration, Office of Acquisition Policy, 2026), the UK Cabinet Office Procurement Policy Note 02/24 (Cabinet Office, 2024a) and Guidelines for AI Procurement (Office for Artificial Intelligence, 2020), the European Union (EU Model Contractual Clauses), and the OECD. Rather than a narrative overview, these instruments were evaluated textually using a deductive comparative framework. This qualitative analysis functions as a conceptual benchmark mapping exercise rather than an empirical statistical coding project. UK materials expect visible transparency disciplines, including Algorithmic Transparency Recording Standard (ATRS) disclosures. EU materials constitutionalise reason-giving and access to the file through Article 41 of the Charter, whilst Article 52(1) requires any limitation to respect the essence of the right (EU Agency for Fundamental Rights, 2026b). Together, these materials show that contracts cannot quietly thin administrative rights below constitutional minimums.

The doctrinal method is anchored by case law acting as constitutional bellwethers. Court of Justice of the European Union (CJEU) and United Kingdom (UK) authorities illuminate limits on opaque, unreviewable, or rights-infringing administration where data protection, transparency, effective explanation, secrecy, and outsourcing converge. This targeted legal corpus incorporates critical public-law, constitutional, and regulatory source materials spanning multiple jurisdictions to address the limits of automated state action, procedural fairness, and non-delegable public duties, including Doody (1994), Smith (2006), Loomis (2016), Miller (2017), UNISON (2017), Pintarich (2018), Privacy International (2019), Hoareau (2020), SyRI (2020), Schrems II (2020), and Liberty (2025). These jurisprudential bellwethers are cross-referenced with selected statutory instruments, transnational human rights frameworks, and public sector accountability regimes that bear directly upon the externalisation of public services, including the Charter of Fundamental Rights of the European Union (2012), the European Commission (2024), Loper Bright (2024), and the Council of Europe (2024). Furthermore, the analysis draws extensively on specialised procurement, data-protection, and anti-corruption authorities that govern accountability failures in outsourced environments, such as NEAT (2003), Belize Alliance (2004), YL (2007), Nash (2013), Steele (2019), R (Good Law Project) (2022a), R (Good Law Project) (2022b), R (Good Law Project and EveryDoctor) (2022), the Public Accountability Bill (2025), and the OECD (2025c). Systematically evaluating this regulatory corpus ensures that the proposed framework remains doctrinally resilient across both common law and civil law jurisdictions.

## 3 Comparative regulatory paradigms and the legality gap

### 3.1 The ontological shift: AIaaS as outsourced administrative capacity

Descriptively, this study conceptualises the integration of AIaaS in government not as a passive tool, but as the contractual migration of administrative intelligence into a proprietary service environment (OECD, 2025b; Yeung, 2018). Normatively, this article argues that because the supplier maintains the model and controls updates, this digital relocation establishes an acute legality gap that requires public law safeguards to be programmatically embedded within procurement terms rather than relying solely on traditional *post hoc* procedural remedies.

The distinction between buying a tool and outsourcing capacity is a distinction in jurisdictional competence. Ordinary software may leave judgement inside the authority; an AI-enabled service that structures triage, eligibility, investigation, ranking, or suspicion displaces practical cognition into vendor architecture. To treat that arrangement as mere assistance understates its public-law significance (Citron, 2008; Mashaw, 1985).

That transfer sharpens the question of digital sovereignty. A state that lacks the contractual right to reconstruct the decision-state has ceded part of its jurisdictional competence. If it cannot recover the relevant model version, metadata, thresholds, explanations, and change history, it cannot govern the evidentiary basis of its own acts. Administrative sovereignty requires the capacity to understand, reproduce, contest, and displace the logic through which public power is operationalised.

The Australian *Pintarich v Deputy Commissioner of Taxation* (Pintarich, 2018) case crystallises the doctrinal paradox with unusual force. The Full Federal Court held that a computer-generated letter produced without human mental input did not amount to a valid legal decision for the relevant statutory purpose (Allars, 2024). On one reading, the case exposes a legal nullity: if no legally cognisable act of mind occurred, then no valid administrative decision came into existence. Yet that formal conclusion collides with the functional reality experienced by the citizen, for whom the automated result was neither abstract nor harmless. It affected legal position, generated compliance pressures, and carried the practical force of state action. *Pintarich* therefore reveals a legal vacuum at the heart of automated governance: a result may be void in doctrine whilst remaining coercive in life. The law’s categories fracture. The citizen still bears the weight (Hildebrandt, 2020). As the Royal Commission into the Robodebt Scheme (2023) demonstrated, the surrender of administrative oversight to automated processes can lead to systemic illegality that escapes detection until the human cost is catastrophic (Services Australia, 2026). This doctrinal fracture was explicitly recognised by Kerr J in his powerful dissent in *Pintarich*. Whilst the majority held that an automated letter did not constitute a valid legal decision absent an official’s conscious internal mental state, Kerr J argued that an administrative ‘decision’ must be defined by its objective and external legal effects on the citizen (Allars, 2024). His Honour warned that requiring a human mental process to trigger judicial review ignores the reality of modern bulk automated decision-making, effectively creating a jurisdictional black hole that immunises algorithmic governance from public law scrutiny.

AIaaS intensifies that paradox because the relevant intelligence may be externally held. The supplier may determine weighting, thresholds, retraining cadence, and disclosure conditions. Formal human approval does not cure structural dependence if the state cannot command the decisional pathway. Under a strategic-actor analysis, the provider may exploit ambiguity in disclosure, silence on reconstruction, and elasticity in update language to convert operational control into *de facto* public power (Craig, 2021; Harlow and Rawlings, 2022).

The common law position now points in the same direction. In *Yorston v Attorney-General* (Yorston, 2026), the New Zealand Court of Appeal rejected the notion that the automated generation of convictions history reports fell beyond judicial review merely because the process was software-mediated. The reasoning matters because it preserves the public character of executive power even where the delivery mechanism is digital, mechanical, or institutionally remote: the Ministry retained control, responsibility, and therefore reviewable public accountability for the system’s operation. Public power does not lose its nature when it passes through a machine. Nor does it lose that nature when the machine is privately supplied (Simpson Grierson, 2026).

The doctrinal pathways for challenging contractual opacity vary significantly by legal system. In common law jurisdictions like the UK and Australia, this conclusion frequently proceeds through *ultra vires* or the unlawful abdication of power. The landmark decisions in Miller (2017) and Fire Brigades Union (1995) and recent algorithmic-administration scholarship point in the same direction: legality requires visible governance chains, a clear statutory basis, explicit decision rules, override structures, and robust change controls (Citron, 2008; Pasquale, 2015; Yeung, 2018). Conversely, in the US federal context, such opacity is more accurately litigated as an ‘arbitrary and capricious’ procedural violation under the Administrative Procedure Act (APA) (1946), rather than a strict jurisdictional *ultra vires* defect. Across these variations, delegation doctrine cannot rest on formal labels where proprietary systems materially structure the reasons, priorities, inferences, and classifications through which statutory powers are exercised. Rubber-stamping an opaque output does not satisfy the requirement for a valid act of mind if the official is contractually barred from the evidence needed for independent judgement (Citron, 2008; Yeung, 2018; Pasquale, 2015; Ameen et al., 2026; Taylor, 2023).

### 3.2 The legality gap in traditional administrative law

The second finding is that traditional administrative law was not designed for decision-making distributed across vendors, data infrastructures, and update pipelines. It presupposes a decision-maker with the record, reasons, disclosure capacity, and evidentiary foundation needed for review. Outsourced AI disrupts that coherence by placing the factual substrate of legality outside the public body whilst leaving formal responsibility with the authority (Citron, 2008; Pasquale, 2015).

The SyRI (2020) litigation in the Netherlands exposed the first rupture. The District Court of The Hague held that the algorithmic welfare-fraud framework violated Article 8 of the ECHR because its interference with private life was insufficiently transparent and foreseeable. This landmark decision demonstrates that opacity in digital governance can itself constitute a systemic legality failure, as citizens cannot contest what they cannot see and courts cannot supervise what remains hidden (SyRI, 2020; see also van Loo, 2019; Public Law Project, 2024). Warren and Brandeis and Nissenbaum reinforce the point: data gathered for one administrative context cannot be repurposed into an opaque engine of suspicion without rupturing contextual integrity (Warren and Brandeis, 1890; Nissenbaum, 2010).

Bridges (2020) exposed a cognate departure from the standpoint of equality and institutional responsibility. The UK Court of Appeal held that the deployment of automated facial recognition was unlawful, because the police force had failed to take reasonable steps to ensure the software was free from unacceptable bias, thereby breaching the Public Sector Equality Duty (Equality Act 2010, 2010, s. 149). The importance of Bridges (2020) lies in its absolute rejection of the idea that vendor assurance can substitute for public inquiry. Under this public inquiry doctrine, a failure to contract for adequate verification mechanisms risks leaving the public body structurally exposed to a breach of its non-delegable duties if systemic biases go undetected (Bridges, 2020; see also Office for Artificial Intelligence, 2020).

Loomis (2016) offers a different but complementary warning. The Wisconsin Supreme Court permitted reliance upon the proprietary COMPAS risk tool in sentencing, albeit accompanied by cautions as to its limits and the explicit instruction not to treat it as determinative. Yet the more enduring jurisprudential significance of the case lies in the structure of the systemic problem it revealed: due-process claims become fragile where an individual is materially affected by an administrative framework whose internal logic is shielded by proprietary protection (Loomis, 2016). Whether one agrees with the outcome or not, *Loomis* exposes the pressure point at which trade secrecy and procedural fairness begin to grind against one another. If neither the individual nor the state can interrogate the basis of a recommendation that shapes public action, the formal availability of review becomes an evidentiary nullity (Harvard Law Review, 2017).

The legality gap emerges from the supplier’s informational advantage, Software-as-a-Service (SaaS) update instability, and responsibility diffused across integrators, cloud providers, data brokers, and model developers. Public accountability remains with the authority, yet the contract may deprive it of the evidence needed to explain, justify, and correct. The deeper problem is self-blinding: failure to secure the evidentiary conditions of public acts at procurement (OECD, 2025b; Pasquale, 2015; Citron, 2008; Public Law Project, 2024).

The significance of that *ex ante* design sharpens further in the emerging post-deference era. In Loper Bright (2024), the United States (US) Supreme Court held that the APA requires courts to exercise independent judgement on questions of statutory authority, clarifying that textual ambiguity does not licence judicial abdication in favour of agency interpretation (Loper Bright, 2024; see also Craig, 2025). The comparative lesson is larger than American administrative law. If courts must not surrender the final word on statutory meaning to agencies, still less may they permit that meaning to be sedimented inside proprietary AI systems whose operational logic is supplied by private vendors. Explainability fidelity is thus not a merely technical aspiration. It is the evidentiary condition of judicial supremacy over statutory meaning. Statutory interpretation is a judicial function, not a computational side effect of a rented model.

### 3.3 Non-derogable duties of good administration in AI-enabled government

The third finding suggests that certain core administrative responsibilities function as non-derogable baselines that public authorities cannot easily outsource. Consequently, under common law principles of non-delegation, procuring a system that structurally prevents the independent exercise of statutory judgement may be vulnerable to challenges of *ultra vires* or unlawful abdication of power. As demonstrated by SyRI (2020) and Privacy International (2019), contractual secrecy that prevents public review functions as a practical ouster of supervisory jurisdiction (SyRI, 2020; Privacy International, 2019; see also Craig, 2021; Harlow and Rawlings, 2022).

The first anchor is article 41 of the Charter of Fundamental Rights of the EU, which requires impartial, fair, and timely handling and protects rights connected to being heard, file access, and reasons. Although addressed to EU institutions, it crystallises a wider constitutional understanding of administrative legality (EU Agency for Fundamental Rights, 2026a). In the UK, the point rests on candour, reasoned justification, equality-conscious inquiry, and responsible ministerial control, especially after retained EU law became assimilated law and the Retained EU Law (Revocation and Reform) Act 2023 (2023) ended EU law supremacy and general principles (Sales, 2026). If those duties are not translated into audit rights, disclosure duties, and override powers, they risk evaporating in digital procurement (Gibson, 2025; EU Agency for Fundamental Rights, 2026a).

The second anchor is data protection law, particularly EU General Data Protection Regulation 2016/679 (GDPR) article 22 and related rights of access and meaningful information about automated processing. Article 22 is not a complete code for public sector AI, nor does it exhaust the requirements of administrative law. Nevertheless, it is significant because it reflects a legislative judgement that solely automated decisions with legal or similarly significant effects require special control (GDPR, 2016, arts 15(1)(h), 22; SCHUFA, 2023). It therefore supports, rather than replaces, the broader proposition that reason-giving and human contestability cannot be treated as optional commercial features where automated systems shape public acts (Edwards and Veale, 2018). That rights-based conclusion is reinforced by Ligue des Droits Humains (2022), in which the Court of Justice accepted that technologically mediated security governance must remain constrained by necessity, proportionality, and strict limits on predictive profiling. The case belongs in this argument because it shows that large-scale algorithmic sorting cannot be normalised merely by invoking administrative utility. Human dignity and privacy are not peripheral values at the edge of the system. They constitute non-derogable constraints that define the constitutional boundaries of lawful digital governance.

Recent case law strengthens that reading. In CK (2025), the Court of Justice held that a data subject must be able to receive meaningful information about the logic involved in automated decision-making sufficient to enable understanding and challenge, clarifying that trade secrecy cannot justify withholding all such information. The ruling should be understood as more than a technical interpretation of access rights (Directive (EU) 2016/943, 2016, arts 5, 9). It operates as a constitutional limit on blanket secrecy where automated evaluation bears upon an individual’s legal position. It substantially weakens the claim that proprietary protection can immunise public-facing decision systems from scrutiny. In procurement terms, that override must be mirrored contractually: clauses that leave the authority without meaningful explanatory access do not merely manage confidentiality badly; they entrench a structure of administration that is incompatible with challenge, candour, and the right to know why power has been exercised in a particular way.

The third anchor is equality law and anti-discrimination obligations. The UK case Bridges (2020) makes clear that a public body cannot rely on a vendor’s assurances as a substitute for its own duty of inquiry. In AI procurement, that means non-discrimination cannot be satisfied by a bare warranty that the system is fair. The authority must have rights to examine performance across relevant groups, require testing against discriminatory effects, and intervene where the system’s operation undermines equal treatment. A contract that permits deployment whilst withholding the data and evidence necessary for such scrutiny is difficult to reconcile with the public body’s continuing obligations (Wachter et al., 2021). The same point has been driven home by critical scholarship. Benjamin shows how apparently neutral digital systems can sediment racial hierarchy into technical routine, whilst Selbst and Barocas demonstrate that demands for explanation are often rendered superficial precisely where socio-technical complexity is invoked to resist accountability. Read against Bridges (2020), their work confirms that a public body must contract for evidence of equal treatment rather than accept vendor narratives of fairness as a substitute for proof (Benjamin, 2019; Selbst and Barocas, 2018).

The fourth anchor is meaningful human oversight. The EU AI Act recognises human oversight as a core requirement for high-risk AI systems and imposes corresponding obligations on providers and deployers (AI Act, 2024, arts 14 and 26; Annex III). Yet the relevant public law question is not exhausted by the presence of a human reviewer somewhere in the workflow. Oversight must be institutionally meaningful. It should involve authority, competence, access to relevant information, and the ability to suspend or depart from system outputs. A human who lacks the contractual rights or technical means to understand the output is not exercising legal oversight in any substantial sense. Oversight without information risks becoming a ritual rather than a safeguard (Hildebrandt, 2020; Yeung, 2018). Because administrative functions such as welfare and migration are designated as high-risk under Annex III of the AI Act, the pre-deployment rationality controls proposed here form part of the *ex ante* compliance logic required for lawful deployment ahead of the revised 2 December 2027 standalone enforcement deadline (AI Act, 2024, arts 9–15, 26, 27; Annex III, points 5 and 7).

The fifth duty is traceability. Administrative review depends upon a record. In AI settings that record must include not only the final output but the relevant model version, the data or metadata necessary to reconstruct the decisional context, the explanations furnished at the time, and the changes introduced through later updates. Without such traceability, appeals, ombuds investigations, audits, and judicial review may proceed on an evidentiary deficit that the authority itself created through weak procurement design. The state’s duty to keep a sufficient record is therefore not displaced by the fact that the system is privately supplied (Public Law Project, 2024; Mashaw, 1985).

The sixth duty is access to remedy. The United Nations Guiding Principles on Business and Human Rights (UNGPs) insist that states should take appropriate steps to ensure that when abuses occur within their territory or jurisdiction, those affected have access to effective remedy, and they emphasise the importance of oversight where public services are outsourced (United Nations, 2011). In the present context, access to remedy requires more than a formal right to complain to the authority after harm occurs. It requires a contractual architecture that preserves evidence, identifies responsible actors, allows meaningful investigation, and supports corrective action without disabling the service altogether. A right that cannot be operationalised against the infrastructural realities of AI procurement is not an effective remedy.

### 3.4 Contract as constitutional instrument

To establish an evidentiary record, this article proposes a model Application Programming Interface (API) gateway architecture where outputs include a structured JavaScript Object Notation (JSON) metadata payload, providing the technical traceability required to support subsequent legal reconstruction. Because internal execution variables are not uniform across all AI architectures, the procurement contract must adjust its telemetry requirements to the specific system type. For example, whilst deep learning architectures may output confidence scores or model hashes, generative models require temperature logs. Where specific parameters are unavailable or technically meaningless within a vendor’s proprietary architecture, the contract should substitute equivalent diagnostic data capable of proving system stability and path verification. At minimum, the payload must record these versioned operational variables in a machine-readable form to satisfy statutory automated logging and public-law disclosure mandates (AI Act, 2024, art 12; Cabinet Office, 2025b; OpenTelemetry Authors, 2026). Consolidating this data into a standardised data-interchange format allows authorities to reconstruct the execution environment at the point of origin:

Example 1. Operational metadata schema for automated reason-giving.

{

“decision_id”: “uuid-v4,”

“inference_timestamp”: “2026–08-04 T02:22:00Z,”

“model_hash_v2”: “sha256:<model-binary-or-checkpoint-hash>”,

“context_window_token_length”: 32768,

“temperature_setting”: 0.2,

“system_prompt_commit_id”: “git:<commit-sha>”,

“input_features_hash”: “sha256:<hash-of-raw-administrative-input-fields>”,

“inference_confidence_score”: 0.87,

“retrieval_corpus_version”: “evidence-pack-2026-06-18,”

“human_override_status”: “not_invoked,”

“audit_trace_id”: “otel-trace-id.”

}

By materialising the abstract public-law duty of candour into an immutable data packet, this technical schema functions as a foundational architecture upon which the broader contract operates as a constitutional instrument. This telemetry does not, by itself, convert an algorithmic output into a legally adequate reason. Instead, it serves as a baseline evidentiary record, capturing the technical provenance required for subsequent human officials to construct a legally sound justification.

It also gives procurement officers a concrete artefact to require in service-level obligations, audit clauses and data-retention schedules, rather than relying on vendor assurances after the legality of a decision has already been contested. This technical metadata serves as an interpretive aid rather than a self-executing validation mechanism; it must be paired with human oversight protocols to provide human auditors with the necessary context to determine whether the system operated within legitimate legal and policy boundaries during that specific decision event. Where human intervention is actively triggered, the payload structure conditionally expands to append the corresponding override_reason_code and escalation_id specified in the oversight matrix (Table 1).

**Table 1:** Procurement compliance control matrix.

Legal duty	Contract clause	Machine-readable artefact	Event log	Review trigger	Responsible actor	Retention period
Reason-giving and explainability	Decision-specific explanation; metadata-retention term.	Explanation schema; model card; decision-state snapshot.	Inference timestamp; model version; input fields; explanation package generated.	Adverse decision; appeal; audit request; material update.	Public authority decision owner; lead vendor.	Life of the decision plus statutory appeal and limitation period.
Non-discrimination and equality	Bias testing; regression testing; protected-characteristic safeguards.	Bias-test report; benchmark dataset register; threshold-control file.	Test run identifier; benchmark version; disparity metrics; remediation action.	Disparate-impact signal; complaints cluster; scheduled revalidation.	Authority equality lead; independent auditor; vendor compliance officer.	Procurement term plus *post*-deployment monitoring period.
Traceability and record preservation	Decision-state reconstruction; immutable logging obligation.	Version-control register; audit log; configuration hash; data-lineage record.	Model release; configuration change; data-source change; override; rollback.	Challenge to a decision; inability to reconstruct system state.	Lead vendor; authority records officer.	Minimum public-records period and any litigation hold.
Update governance and continuous legality	No material update without approval; sandbox testing; legal sign-off.	Change-impact assessment; test certificate; deployment approval record.	Patch; retraining cycle; orchestration change; threshold change.	Material behavioural change; update affecting statutory criteria.	Vendor release manager; public authority senior responsible owner.	Entire service term plus audit and appeal window.
Human oversight and intervention	Meaningful human review; override right; escalation right.	Oversight workflow; escalation register; override reason code.	Automated recommendation; human acceptance; override; escalation; outcome.	High-impact classification; contested output; low-confidence recommendation.	Trained public official; vendor technical support.	Life of decision plus appeal period.
Remedial access and correction	Correction cost model; re-adjudication support obligation.	Affected-file register; remediation-cost calculation; forensic report.	Systemic error; missing metadata; unlawful output; manual re-adjudication action.	Loss of auditability; confirmed legality failure; court or tribunal review.	Public authority remediation team; vendor; independent auditor.	Until all affected files are corrected and review routes expire.
Exit continuity and sovereign control	Unilateral Termination for Legality; neutral transition protocol.	Data-export package; interoperability map; deletion certificate; transition plan.	Termination trigger; export initiation; transfer completion; deletion confirmation.	Legality failure; conflict of interest; vendor non-compliance; lock-in risk.	Contracting authority; successor provider; incumbent vendor.	Transition period plus statutory record-retention period.

If those duties are non-derogable, the public contract becomes a constitutional instrument in functional terms. It is not constitutional law, but it determines whether public-law duties can be made effective once administrative capacity is externalised. By allocating control over information, intervention, documentation, updates, audit access, liability, and exit, it determines whether the authority can comply with duties that already bind it (Harlow and Rawlings, 2022; Craig, 2021).

The point is easiest to see by contrast with ordinary commercial contracting. Commercial contracts often focus on availability, service levels, indemnities, and price. Those matters remain relevant in public AI procurement, but they are insufficient. A perfectly available system may still be unlawful if it cannot provide reasons, if it entrenches discrimination, if its updates alter legal treatment without approval, or if neither the authority nor an affected person can reconstruct what happened in a contested case. In such settings, legality must become an object of contractual design. The issue is not merely service performance, but the preservation of the conditions under which administrative justice can still operate (Office for Artificial Intelligence, 2020; European Commission, 2025b).

To make oversight enforceable, contracts should define machine-readable governance interfaces, secure APIs, and standardised JSON logging schemas for querying decision parameters. They should impose decision-specific explainability, preserve versioning and metadata, regulate updates through testing and approval, and specify correction, suspension, transition, and redress pathways. The authority must not remain legally accountable whilst being contractually denied the information needed to discharge that accountability (European Commission, 2025b; Public Law Project, 2024).

The language of constitutionalisation is useful if kept precise. Procurement determines whether outsourced administrative capacity remains contestable, reviewable, and interruptible by law before the system shapes decisions. Schrems II (2020) supplies the analogy: formal safeguards are insufficient where rights leak away in practise; supplementary measures are required. AI procurement likewise requires enhanced audit rights, anti-secrecy overrides, update sandboxing, escrowed documentation, and decision-state reconstruction rights to prevent legality leakage between a public authority and a private supplier (Citron, 2008; Hildebrandt, 2020; Cabinet Office, 2025b).

The operationalisation of this non-delegable core requires a nested contractual structure modelled within the Three-Layer Accountability Framework introduced in Section 1 (see Figure 1). The framework is consolidated under three core pillars: Evidentiary Sovereignty (operationalised via transitive audit rights), Continuous Legality (enforced through Unilateral Termination for Legality (UTL) and transition protocols to prevent administrative amnesia; Brauneis and Goodman, 2018), and Redress Scaling (executed through a programmatic correction cost model (CCM) formula). The enforceability of this indemnity is anchored in a modern interpretation of the penalty rule, giving weight to the state’s legitimate interest in administrative integrity (Leung, 2017; Rowan, 2019). The complete legal specifications implementing these mechanisms are compiled within the Supplementary material 1. Whilst this strict non-derogable core safeguards public law baselines, its rigid auditing frameworks introduce compliance overheads and participation barriers for small and medium-sized enterprises (SMEs). This structural tension provides the normative yardstick to measure the current ‘contractual vacuum’ across the EU, UK, and OECD procurement regimes evaluated in the following section.

**Figure 1:**
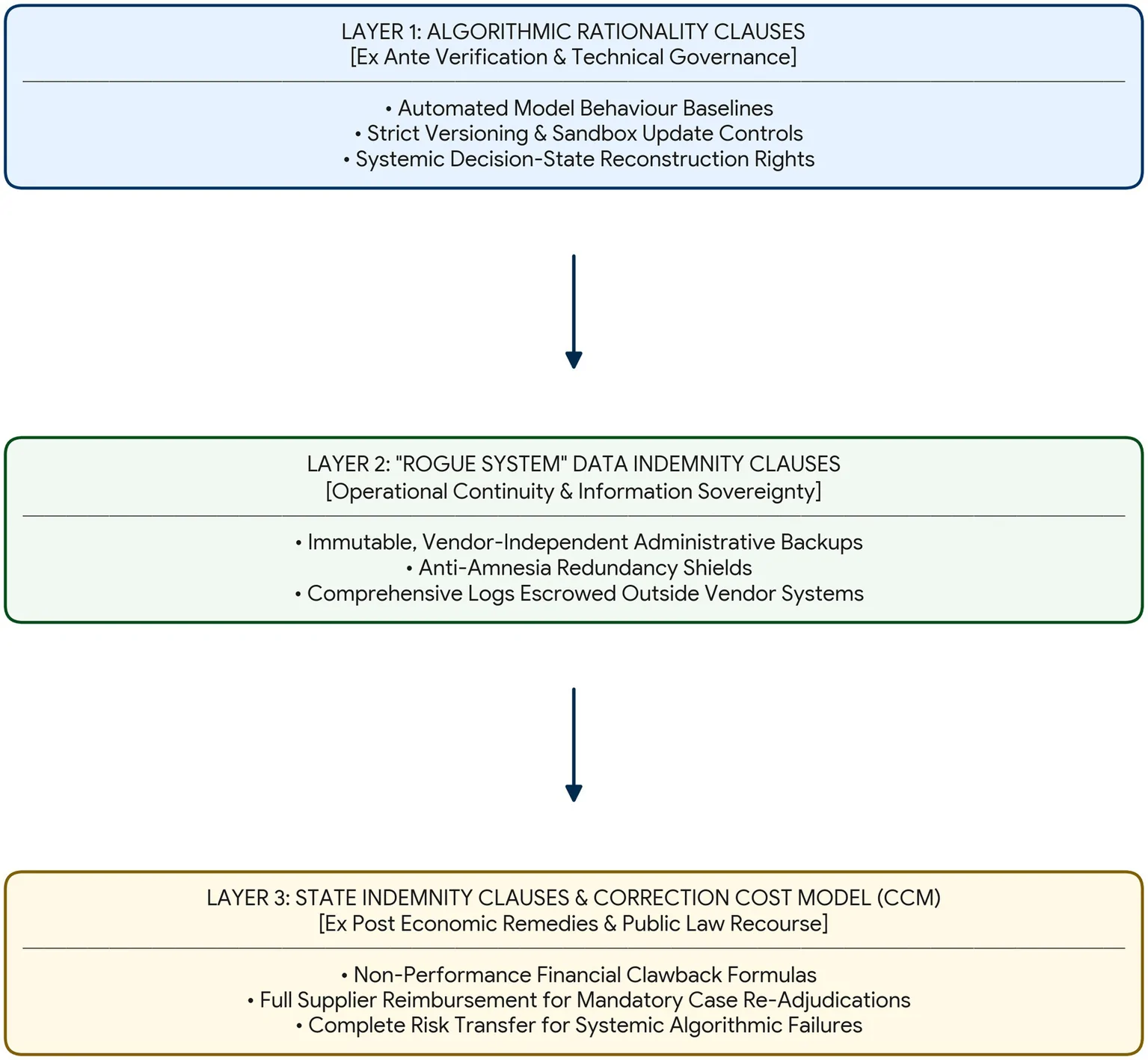
The structural architecture of the Three-Layer Accountability Framework in public sector AI services. The nested model establishes a sequential progression from *ex ante* algorithmic verification controls through to *ex post* financial remedies designed to preserve non-delegable administrative duties.

### 3.5 Comparative analysis: EU, UK, and OECD frameworks

This section analyses the divergent approaches of the EU, UK, and OECD to determine how they operationalise—or fail to operationalise—the non-derogable core of good administration. The EU provides the most elaborate regulatory anchor. These specialised rules function alongside the baseline statutory framework governing public contracts across the single market (Directive 2014/24/EU, 2014). Specifically, Regulation (EU) 2024/1689, arts. 14, 26, and Annex III adopts a risk-based structure and imposes specific obligations for high-risk AI systems, including requirements relating to risk management, data governance, technical documentation, record-keeping, transparency, human oversight, accuracy, robustness, and cybersecurity (AI Act, 2024, arts 8–15; Annex III). The AI Act is not itself a procurement code, but it matters directly for public contracting because many governmental uses of AI will fall within or adjacent to its high-risk categories, and because deployer obligations make clear that legal responsibility does not stop at purchase. Particularly relevant are the provisions on human oversight and deployer responsibilities, which reinforce the proposition that public authorities cannot discharge legality merely by relying on provider compliance (AI Act, 2024, arts 14 and 26; Annex III).

The updated EU model contractual clauses for AI procurement build on that regulatory foundation. Their significance lies not simply in their content but in their existence. They represent an official recognition that public buyers need contractual instruments capable of translating AI governance requirements into purchasing practise. The 2025 update distinguishes between a full version for high-risk AI, a light version for non-high-risk AI, and commentary on use and customisation (European Commission, 2025b). That architecture creates the clearest empirical contrast in the comparative materials.

The Full version hardens accountability into evidence by requiring the supplier to provide compliance information, cooperate in audits and inspections, expose risk-management systems, make staff available for interview, and allow access to relevant locations (European Commission, 2025a). It also provides reporting, defect remediation, periodic audits, and fresh audit rights after substantial modification. The Light version preserves a compliance obligation and a duty to provide information, but omits comparable inspection, interview, location-access, report-publication, and recurring-audit machinery (European Commission, 2025a). The difference is juridically important: evidence becomes an enforceable entitlement in the Full version but a more fragile assurance in the Light version, despite the OECD’s 2025 emphasis on end-to-end transparency (The Global Partnership on Artificial Intelligence, 2024; European Commission, 2025a). Table 2 compares the EU Model Contractual Clauses Full and Light versions and highlights their constitutional significance for public-sector AI procurement.

**Table 2:** Comparison of the EU model contractual clauses (MCC) full and light versions and their constitutional significance.

Feature	Full version (high-risk)	Light version (non-high-risk)	Constitutional significance
Audit rights	Direct access to locations, staff interviews, and risk-management systems.	General duty to provide information necessary to demonstrate compliance, but explicitly withholds dedicated operational audit and inspection machinery.	Evidentiary sovereignty: Full version treats evidence as a contractual entitlement; Light version thins it into assurance.
Modification/Updates	Mandatory fresh audit and documentation for ‘substantial modifications’.	Standard notification of changes with general compliance warranty.	Continuous legality: Full version prevents ‘silent retraining’ from altering the legal standard without oversight.
Explainability	Decision-specific explanatory requirements tailored to high-risk impacts.	General transparency requirements regarding system capabilities.	Reason-giving fidelity: Ensures that the ‘vendor-controlled decisional infrastructure’ can support the authority’s duty to provide reasons for specific acts.
Human oversight	Detailed requirements for authority, access, and ability to override outputs.	General requirement for human presence in the workflow.	Meaningful review: Prevents ‘automation bias’ by ensuring the human operator has the technical means to interrogate the output.

The comparative analysis reveals three distinct patterns of contractual failure. The first is an information failure, in which visibility into system logic, documentary evidence, and decision-state reconstruction remains partial or conditional. The second is a control failure, in which update governance, human oversight, and intervention powers are acknowledged in principle but insufficiently operationalised in the contract (Pasquale, 2015; Yeung, 2018). The third is a remedial failure, in which rights of challenge are not matched by reliable evidentiary access, continuity safeguards, or practical exit pathways when the system becomes legally unsafe. Across the examined frameworks there is therefore partial convergence on the rhetoric of accountability, but uneven translation of that rhetoric into enforceable contractual architecture, as vividly demonstrated by the EU’s decision to withhold dedicated audit machinery from its Light framework (European Commission, 2025a), a structural compromise mirrored across contemporary international baseline standards (Cabinet Office, 2025a; Cabinet Office, 2026; OECD, 2015, 2025c).

### 3.6 Trade secrets, confidentiality, and the evidentiary crisis

A persistent obstacle to lawful AI-enabled administration is the tension between public-law transparency and private-law secrecy. Providers may invoke trade secrets, Intellectual Property (IP), cybersecurity, or confidentiality to restrict access to code, training data, model logic, or implementation detail. Some confidentiality is legitimate, but it cannot be structured to disable reason-giving, equality testing, or review (Pasquale, 2015; Public Law Project, 2024). Zuboff and O’Neil show why opacity is economically and administratively attractive; procurement must therefore make visibility cheaper than concealment (O’Neil, 2016; Zuboff, 2019).

*Loomis* (Loomis, 2016) is again instructive because it exposes the due process cost of opacity where a proprietary system materially shapes public decision-making. Yet the point now lands with far greater force in European law. In CK (2025), the Court of Justice substantially limited the blanket trade-secret defence by making clear that rights to meaningful information in the context of automated evaluation cannot be reduced to empty formulae through an invocation of secrecy as a blanket obstacle. Read with article 41’s guarantee of good administration and access to the file, the judgement does more than trim an overbroad confidentiality claim. It establishes a hierarchy of norms. In the context of EU data protection law, this jurisprudence suggests that trade secrecy cannot completely override transparency rights, indicating that procurement terms should balance these competing interests. The hierarchy between public transparency and trade secrecy is heavily regime-dependent, meaning this priority cannot simply be mandated uniformly in procurement form. Within the EU, jurisprudence under the AI Act and GDPR increasingly curtails commercial secrecy to protect fundamental rights, suggesting that a clause letting a vendor hide the procedures actually applied in a rights-affecting system threatens legality’s evidential foundation. Conversely, US courts interpreting the Freedom of Information Act (FOIA) Exemption 4 (1966) historically provide robust protections for trade secrets, meaning transparency priorities must be carefully balanced against statutory IP shields on a case-by-case basis rather than forcing an absolute contractual denial of secrecy shields (Critical Mass Energy Project v Nuclear Regulatory Commission, 1992; Food Marketing Institute, 2019).

The evidentiary problem is broader than the disclosure of code. Courts and review bodies often do not need full publication of source code to assess legality. What they need is decision-specific explanatory material, access to relevant documentation, preservation of model-state information, and a controlled mechanism for deeper inspection where necessary. This suggests that the public law response should not be framed as an all-or-nothing dispute between total transparency and absolute secrecy. The more promising approach is a layered access structure. It should include decision-specific reasons for affected individuals, broader documentary access for the authority, secure audit rights for independent experts and oversight bodies, and escrow or protected disclosure mechanisms for litigation or high-level review (Public Law Project, 2024; Pasquale, 2015).

Contract law is central to this layered structure because without express rights the authority may find itself unable to secure the necessary evidence at the moment of dispute. Public interest override clauses should therefore specify that trade secret or confidentiality claims cannot be asserted against the authority, a competent court, an ombuds institution, or an authorised independent auditor where access is necessary to investigate legality, discrimination, or rights impacts. Such clauses need not destroy proprietary interests. They can preserve confidentiality vis-à-vis the general public whilst ensuring controlled disclosure to the actors responsible for review. What they prevent is a more troubling position: one in which the state contracts itself into ignorance (Craig, 2021; Hildebrandt, 2020).

This matters not only for appeals after an adverse decision, but for legality more generally. A decision-maker who cannot access the basis of an automated recommendation may have failed to take relevant considerations into account. A department that cannot test equality impacts may fail to satisfy its public duties. A review body that cannot obtain the relevant system state may be unable to provide effective remedy. In each case the problem is not that public law has relaxed its standards. It is that private contractual arrangements have foreclosed the evidence needed to apply them. The contract should therefore be drafted on the premise that commercial confidentiality yields, in controlled form, to administrative legality (CK, 2025; Bridges, 2020).

### 3.7 Continuous legality: updates as regulatory change

The Continuous Verification Pipeline should therefore be specified as an automated Continuous Integration/Continuous Deployment (CI/CD) Legal Compliance Pipeline and legal sandboxing mechanism (Humble and Farley, 2010). Before any model, prompt, retrieval corpus or decision-rule update is deployed, the vendor should sandbox the candidate release and run automated regression testing script arrays against a fixed, digitally versioned and legally curated baseline dataset of edge-case historical administrative disputes. This automated legal regression testing acts as a mechanism for technical traceability and evidentiary reconstruction rather than a definitive judge of substantive legality; it tracks computational variances over time to ensure technical consistency, but it cannot evaluate open-textured legal standards such as proportionality, equity, or fairness. Instead, it compares explanation fidelity and outcome distributions against baseline configurations to alert human operators of hidden behavioural changes, halting a production release where the baseline data drift threshold exceeds pre-agreed tolerance limits (Gama et al., 2014; OpenTelemetry Authors, 2026; European Commission, 2025b; OECD, 2015).

AIaaS introduces a challenge that ordinary administrative law and ordinary procurement law have not fully absorbed: legality is not fixed at the point of award. A continuously updated service may alter model behaviour, variable weighting, threshold sensitivity, or interaction design after deployment. In commercial settings such updates are routine. In administrative settings they may amount to a practical modification of how legal standards are applied to individuals. The issue is not whether the software has changed, but whether the administration has changed without the legal and procedural controls that such change would ordinarily attract (Yeung, 2018; OECD, 2025b).

Consequently, technical updates must be treated as events of legal significance. An update that changes a system’s evaluation of evidence, recalibrates risk scores, alters triage hierarchies, or modifies recommendation vectors impacts rights and interests no less than a formal policy revision. Yet, conventional SaaS agreements frame these modifications as ordinary maintenance, reserving unchecked technical discretion to the provider. Such frameworks are irreconcilable with foundational principles of public law; if a commercial vendor can unilaterally alter the operational mechanics of an administrative service, the public authority effectively abdicates control over how its own statutory powers is exercised (Office for Artificial Intelligence, 2020; European Commission, 2025b).

To address this ongoing drift, legality must become an architectural release gate: a failed legal regression test triggers an automated deployment block that forces formal administrative review, mimicking security escalation protocols in conventional Development and Operations (DevOps) practise. Concurrently, the public-law concept of sandboxing is clarified as controlled pre-production verification against lawful decision criteria, atypical boundary conditions and equality-relevant cohorts. By embedding continuous compliance monitoring directly into the software release lifecycle, this architecture provides a dynamic solution to the specific systemic volatility of modern cloud deployments. The framework therefore maintains a sharp analytical boundary: technical traceability tools provide the evidentiary foundation for review, but the final determination of substantive legality and compliance with statutory mandates requires contextual human legal judgement.

Such a requirement does more than improve software governance. It preserves the authority’s capacity to determine whether the service continues to operate within the bounds of its legal mandate. It also clarifies accountability when harm occurs. If the provider has altered the system without disclosure or approval, the resulting maladministration can be attributed to a breach of a clearly defined contractual duty. Without such clauses, update-related legality failures may be absorbed into general service risk, leaving affected persons with little visibility into how or why administrative treatment changed over time (Public Law Project, 2024; Hildebrandt, 2020). The point is illuminated by the landmark decision in Secretary of State for Work and Pensions v Johnson (2020), where the UK Court of Appeal held that the operation of the Universal Credit calculation rules was irrational as applied to claimants paid on a recurring but non-monthly cycle. What the case exposes, in present terms, is that the rigidity of the machine is not a defence to administrative unfairness. A welfare architecture does not become lawful merely because its logic is systematic, repeatable, or embedded in code. Where automation crystallises an irrational pattern, the state must correct the system rather than hide behind its own technical inflexibility.

Continuous legality also has implications for record-keeping. It is not enough to preserve the model in its current state. The authority must be able to reconstruct the model state that existed when a particular decision or recommendation was generated. That requires version control, retention of relevant metadata, preservation of explanatory artefacts, and the ability to retrieve a decision-state snapshot within a reasonable period for review or litigation. Absent such arrangements, even a conscientious authority may be unable to answer the basic public law question of what system was used, in what state, and on what basis, when a particular person was affected (Mashaw, 1985; Public Law Project, 2024). This evidentiary baseline directly tracks the high standard of record-keeping expected by courts when evaluating fast-moving administrative triage operations (R (IA and others), 2023).

### 3.8 Remedies and enforcement architecture

The strategic risk in AI procurement is not only obscured system logic but the loss or corruption of evidentiary records on which lawful administration depends. The PocketOS database deletion incident, in which a Claude-powered AI agent wiped a firm’s production infrastructure (The Guardian, 2026), illustrates the risk of administrative amnesia in proprietary Large Language Model (LLM) environments. A public authority neglecting to arrange for redundant backups and system-state recovery creates structural vulnerabilities that directly impede compliance with public record-keeping obligations (Brauneis and Goodman, 2018).

The final major finding is that rights-preserving clauses are insufficient unless paired with an operationalised remedial architecture. As detailed in the Model Clause Blueprint (see Supplementary Material, Clauses 2 and 3), translating public law duties into institutional traction requires explicit contract remedies—including UTL and a formulaic CCM indemnity. By defining these mechanisms as normative design concepts within the procurement lifecycle, the public-law social contract can be structurally maintained across outsourced digital environments.

To achieve this, the first practical component is comprehensive audit access, operationalised in Clause 1 of the Model Clause Blueprint (Transitive Audit and Forensic Access Right). A public authority must maintain contractual rights to inspect technical and organisational materials reasonably necessary to verify performance against legality-sensitive obligations. That includes documentation of training sources, validation methodologies, bias testing, incident logs, change logs, and records of known limitations. Independent audit rights are particularly important where the authority lacks in-house technical expertise. The landmark decision in Bridges (2020) demonstrates exactly why public bodies cannot blindly rely on vendor assurances without independent verification; the contract must therefore ensure that practical verification remains possible throughout the software lifecyle (Office for Artificial Intelligence, 2020).

The second component, explainability and traceability, requires providers to generate and preserve decision-specific explanatory metadata supporting reason-giving, internal review, and external challenge. This obligation must mandate strict retrieval timelines; information must be available swiftly enough to inform appeal, review, ombuds, or court processes. Providers should also maintain secure escrow arrangements preserving the exact system states needed for expert inspection. Crucially, the goal is not transparency for its own sake, but maintaining an evidentiary pathway to test legality (Public Law Project, 2024; AI Act, 2024, arts 12, 14 and 26; Annex III).

The third component is public interest override and controlled disclosure. Confidentiality clauses must not bar access by competent review actors. Contracts should specify disclosure hierarchies, confidentiality undertakings, and secure inspection procedures to protect both proprietary interests and public law values, preventing commercial secrecy disputes from stalling legality proceedings.

The fourth component involves operational step-in rights. Effective step-in necessitates full access to the complete ‘model-environment’—including the software stack, libraries, and internal credentials—rather than just raw model weights. Without this forensic depth, step-in rights remain a hollow legal fiction, leaving the state unable to execute controlled interventions or emergency system replacements.

The fifth component is redress. The UNGPs mandate practical, accessible remedies (United Nations, 2011). AI-enabled administrative contracts should provide clear complaint channels, preserve evidentiary pathways, and support remedies for affected persons. Jurisdictions might adopt third-party beneficiary models giving citizens direct rights to enforce reason-giving clauses, or utilise authority-administered redress schemes funded by the provider following systemic failures (United Nations, 2011; Danish Institute for Human Rights, 2020).

Together, these elements form a non-derogable contractual core. Whilst not exhaustive, this framework establishes the minimum architecture required to prevent the state from outsourcing administrative capacity whilst retaining merely symbolic accountability. Absent this core, privatised digital administration risks converting legally protected rights into empty organisational aspirations (Harlow and Rawlings, 2022; Craig, 2021).

### 3.9 Sovereignty, termination, and conflict of interest

Evidentiary Sovereignty requires contractually protected exit mechanisms (Khan, 2019), operationalised via Clause 2 of the Model Clause Blueprint (UTL). This framework consolidates a unilateral termination right for legality failures, a platform-agnostic data transfer protocol, and an asset-register disclosure script.

Standard software agreements often enforce lock-in through proprietary schemas and infrastructure dependencies. To prevent infrastructure capture, Clause 2.2 mandates a computational Neutral Transition Protocol. Upon a termination trigger, vendors are legally and computationally bound to export all historical transaction logs, versioned schemas, and decision-state metadata in a schema-validated, platform-agnostic format like JSON or Apache Parquet (Opara-Martins et al., 2016; OECD, 2021). This architecture ensures the state can instantly port its administrative footprint to alternative containerised environments without losing operational continuity or disrupting audit trails.

Furthermore, administrative sovereignty requires a proactive framework to manage the corporate political economy of dual-use AI (Khan, 2019; European Commission, 2024). Because algorithmic infrastructures are multi-domain assets, the same underlying models used for domestic civilian triage can be fine-tuned for foreign military deployment, kinetic targeting, or surveillance intelligence (Boulanin and Verbruggen, 2017; NATO, 2024). This fluidness risks procurement capture, data weaponisation, and institutional compromise if public sector data or workflows are exposed to secondary military environments without explicit disclosure (Khan, 2019). The contract must therefore mandate corporate transparency regarding all dual-use, defence, or foreign intelligence contracts. By linking automated notification scripts to the vendor’s enterprise asset register, the state can instantly detect high-risk alignments and trigger an immediate, non-penalising contractual exit before administrative functions become entangled with geopolitical warfare architecture.

## 4 Policy and practise synthesis

Translating the structural legality gap established in Section 3 into actionable design requires an operational framework. The Three-Layer Accountability Framework, summarised by contractual safeguards across outsourced administrative categories in Table 3 and see Appendix, outlines how to restore intelligible, reviewable public power.

**Table 3:** Summary of contractual protections for privatised administrative capacity.

Public function category	Example AI use case	Required contractual safeguards (minimum)
High impact (rights-determinative)	Welfare triage; fraud detection; border screening.	Full transitive audit; decision-state reconstruction; third-party beneficiary rights; administrative liquidated damages.
Medium impact (resource allocation)	School placement; healthcare prioritisation; urban planning.	Algorithmic rationality clause; mandatory update sandboxing; data portability on exit.
Low impact (advisory/internal)	Automated FOI routing; internal resource scheduling.	Version control; transparency logging; standard commercial indemnity.

Ultimately, AIaaS is jurisprudentially vital because it relocates the operational constraints of public administration into commercial contracts, turning procurement into a constitutional control point. Because administrative law demands systemic capacities for explanation, verification, correction, and review, failing to secure these anchors at the point of award erodes downstream rights. The contract becomes the definitive instrument through which public legality is either operationalised or abandoned (Hildebrandt, 2020; Yeung, 2018).

Whilst this accountability mandate is universal, regional enforcement methods diverge. The EU mandates prescriptive, rights-based obligations through the AI Act. Conversely, the UK pursues a regulator-led, principles-based framework governed by common-law evolution and the Procurement Act 2023 (2023) and SureCloud (2025), whilst the US relies on stringent *post*-deference judicial scrutiny and constitutional due process protections. These distinct global paradigms dictate how outsourced public capabilities are bound to the rule of law.

### 4.1 The three-layer accountability framework

Within the third layer, the CCM should be implemented as an active, programmatic, smart-contract-adjacent monitoring script rather than a merely retrospective damages clause. The script continuously inspects operational event logs and audit traces, automatically calculates affected decisions, estimates human review hours via pre-agreed administrative rates, and appends digital administrative damages to the vendor account upon detecting a qualifying legality failure (OpenTelemetry Authors, 2026; Gama et al., 2014). This design preserves human legal judgement whilst automating scale-ready redress.

The transition to a M2M and AI Agent Economy shifts administrative delegation paradigms. Public functions are increasingly executed via autonomous agent swarms optimising decisions in real-time. This “distributed administrative agency” compounds privatisation risks, shifting the locus of administrative cognition from a single model to a transitive chain of interconnected private actors (see Figure 2). Audit traces and remedial duties flow downward along this delivery chain, whilst compliance evidence and explanatory reasons return upward to safeguard public authorities and individuals.

**Figure 2:**
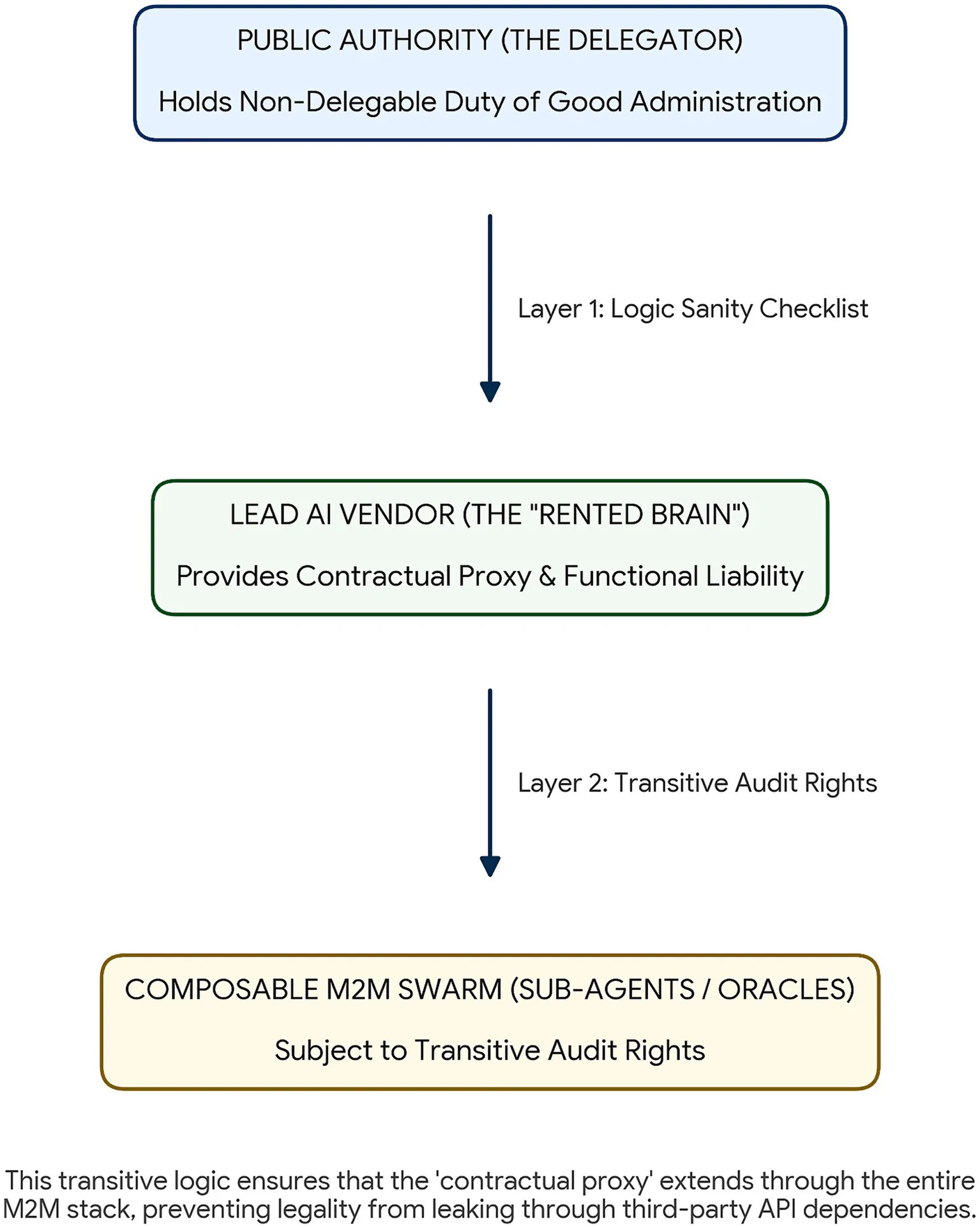
Logic-chain of transitive accountability in machine-to-machine (M2M) governance. The schematic maps the downward delegation of duties from the public authority through the lead AI-as-a-Service vendor to cloud providers, subcontractors, and autonomous sub-agents, alongside the upward return of evidence, reasons, and remedies to the authority and the affected individual.

Whilst introductory frameworks like the EU Model Contractual AI Clauses establish basic compliance boundaries, code-level transitive audit obligations and telemetry-driven liquidated damages do not currently exist in standard procurement practise. Instead, they are introduced here as a novel normative architecture demonstrating how public law verification programmatically scales (Craig, 2021; Public Law Project, 2024), summarised across risk-calibrated deployment tiers using the explicit Required Contractual Safeguards (Minimum) detailed in Tables 3, 4 and see Appendix.

**Table 4:** The three-layer accountability framework for AI-as-a-Service.

Layer	Contractual mechanism	Constitutional function	Trigger condition
1. Pre-deployment (validation)	Algorithmic rationality clause and transitive audit.	Evidentiary sovereignty: Ensures the state can verify the decisional logic and evidentiary basis of the system.	Prior to go-live; triggered by any material update to model behaviour, thresholds, orchestration logic, data sources, or underlying model configuration.
2. Operational (liability)	Unilateral termination for legality (UTL); Secure forensic access.	Continuous legality: allows the state to terminate vendor-controlled decisional infrastructure without commercial penalty.	Identification of discriminatory output, unexplainable logic, or vendor military dual use.
3. Restitutive (repair)	Refined correction cost model (CCM) Formula (Equation 1) and Rogue data indemnity.	Reasoning fidelity and redress: Converts loss of reconstructability into a financial liability.	Systemic failure, unauthorised data deletion, or ‘rogue’ logic execution requiring manual re-adjudication.

The third layer operationalises recovery through the Refined CCM, a programmatic liquidated damages formula integrated directly into the procurement contract. Triggered automatically by an unapproved software state or unnotified model drift recorded in system logs, the model calculates total remediable damages via Equation 1:


D_total=(H_staff×R_overhead)+C_sandbox+F_penalty (1)

Where D_total is total liquidated administrative damages; H_staff represents staff remediation hours; R_overhead is the standardised public agency hourly overhead rate; C_sandbox is the fixed computational cost of resetting the legal sandbox; and F_penalty is the pre-agreed contract baseline penalty fee for unapproved variance.

For a concrete worked example, if an unnotified vendor update causes logic drift requiring 40 h of personnel time (H_staff = 40) at an overhead rate of $150 per hour (R_overhead = 150), combined with a flat $2,000 computational sandbox reset cost (C_sandbox = 2000) and a pre-agreed baseline penalty of $50,000 (F_penalty = 50,000), the programmatic calculation yields:


(40×$150)+$2,000+$50,000=$58,000 (2)

By demonstrating that the state possesses a legitimate, non-commercial interest in preserving administrative integrity, this design supports characterising the CCM as a proportionate liquidated damages mechanism rather than an unenforceable penalty, shifting forensic costs back to the contractor (Leung, 2017; Rowan, 2019). This directly aligns economic risk with administrative duty.

These risk categories are heuristic and subject to “contextual elasticity.” For instance, healthcare resource allocation categorised as “Medium Impact” escalates to “High Impact” where decisions implicate the non-derogable Right to Life or sensitive biometric “Oracles.” Because the boundary between administrative utility and fundamental rights is fluid, authorities must retain discretion to up-calibrate safeguards based on constitutional weight over commercial convenience (Hildebrandt, 2020; Yeung, 2018).

Consequently, migrating public power into proprietary infrastructures creates friction between constitutional demands and vendor commercial shields. This is illustrated by the UK’s procurement strategy for National Highways, which contracted providers like Deloitte to scale its Chief Data Office and deploy predictive data capabilities (Safer Highways, 2025; Cabinet Office, 2025b; Government Digital Service, 2025). Because negotiated public agreements are routinely withheld under commercial confidentiality, evaluation must analyse official public model frameworks alongside standard commercial defaults to map systemic parameters (Piggott, 2026; Cabinet Office, 2025b).

Contemporary public sector templates reveal that official frameworks are explicitly designed to counteract standard commercial defaults and liability limitations. For example, the EU Model Contractual AI Clauses (MCC-AI) impose strict data governance and transparency baselines for high-risk systems to balance standard master service agreements where dominant providers like Google Cloud (Google Cloud, 2020; European Commission, 2025b) or OpenAI (OpenAI, 2025; OECD, 2025a) disclaim statutory warranties. Similarly, the US General Services Administration, Office of Acquisition Policy (2026) basic safeguarding directives and the UK Cabinet Office Procurement Policy Note (PPN 02/24) (Cabinet Office, 2024b) systematically attempt to override standard vendor liabilities – such as Microsoft’s reverse-engineering prohibitions (Microsoft, 2026; Office for Artificial Intelligence, 2020) or universal enterprise service-credit caps (Softchoice, 2025; OECD, 2025a) – by mandating proactive supplier disclosures. Assuming these commercial tensions persist in customised public agreements, the resulting conceptual mismatch is summarised in Table 5 (The Conceptual Clash), with its technical transposition compiled in the Procurement Compliance Control Matrix (Table 1).

**Table 5:** The conceptual clash: public law norms vs. commercial AIaaS defaults.

Public administrative law norm	Standard commercial AIaaS contract default	The proposed contractual solution
Duty of legality and non-bias	Disclaimed: vendor disclaims all warranties of fitness for a specific purpose or statutory compliance.	Unilateral termination for legality (UTL) triggers contract cancellation if bias is found.
Duty of candour/reasons-giving	Obfuscated: proprietary code, trade secrets, and IP clauses block algorithmic inspection.	Transitive forensic access mandates secure technical disclosure sufficient for independent verification as a performance condition.
Restitutive justice/remediation	Capped: liability limits vendor exposure to a few months of service credits (refunds).	Correction cost model (CCM) forces vendor to fund the manual re-adjudication of files.
Sovereign administrative control	Externalised: vendor can push silent software updates or change inferential logic at will.	*Ex ante* validation requires strict testing before any code shift goes live.

### 4.2 Procurement compliance control matrix

The structured transposition of these non-derogable public law values into distinct technical and organisational safeguards is systematically mapped out in the Appendix and Table 1. This matrix explicitly matches core Legal Duties with their corresponding Contract Clauses, Machine-Readable Artefacts, Event Logs, Review Triggers, Responsible Actors, and Retention Periods to guide procurement officers during active contract negotiation.

### 4.3 Actionable recommendations

The Transitive Audit, UTL, and CCM mechanisms should be deployed as risk-calibrated tools securing administrative compliance at contractual origin, operationalised through the full model clauses in the Supplementary material 1.

Translating this framework into procurement design requires addressing commercial incentives. From a Holmesian strategic-actor perspective, profit-seeking vendors exploit contractual silence to maximise leverage: loose data-reuse language invites training expansion, weak deletion duties expand retention, and low liability caps externalise the costs of administrative repair (Holmes, 1897; Craig, 2021; Lifshitz, 2023; Mack, 2025). This risk is acute where public records are reused in dual-use secondary markets. If vendors span civilian and defence sectors, sensitive state data requires explicit use, segregation, deletion, and audit restrictions to prevent unapproved model fine-tuning beyond domestic statutory mandates (Khan, 2019; Zuboff, 2019).

Conversely, a Dworkinian lens demands institutional integrity, treating the contract as a constitutional site where candour, equality, and the rule of law operate as non-negotiable architectural boundaries. Hard-edged clauses must replace vague reasonable-endeavours language, soft cooperation formulas, and supplier-defined documentation standards, securing decision-state reconstruction, model-version histories, and liability calibrated to the cost of legality failures.

Whilst courts remain cautious in designating private contractors as functional public authorities under Section 6 of the UK Human Rights Act 1998 (YL, 2007), embedding these obligations within service-level agreements transforms vendors into contractual stewards of administrative justice (Donnelly, 2007). The state cannot lease decisional machinery and plead contractual distance when legality fails. As reaffirmed in R (Good Law Project) (2022a), the high degree of transparency required by public law is a mandatory baseline that contracts cannot diminish; failure to draft for legality constitutes a failure of public law design (Pasquale, 2015; Clayton, 2015).

Finally, enforcing these provisions requires navigating localised third-party contract rules. Whilst incorporating public interest clauses explores how third-party beneficiary frameworks support citizen-led enforcement, US plaintiffs face high barriers due to a strong judicial presumption against third-party status in government contracts (Restatement (Second) of Contracts, 1981). Conversely, the UK’s Contracts (Rights of Third Parties) Act, 1999 offers a more viable pathway if express contractual intent exists (Macmillan, 2000), though future statutory frameworks should ideally confer third-party standing by operation of law (Sanchez-Graells, 2025). Where feasible, this integration provides citizens with a direct juridical hook for disclosure and challenge support without collapsing public law into private law (Uysal, 2024; United Nations, 2011; Public Law Project, 2024).

Ultimately, digital constitutionalism demands structured visibility over *post hoc* reassurance (Public Law Project, 2024; European Commission, 2025b).

## 5 Discussion and limitations

Contractual constitutionalism is not self-executing. Public authorities may hold extensive audit rights but lack the technical skill, tooling, secure environments, or budgets required to enforce them, preserving a conceptual pathway to legality rather than operational reality. Mistaking the possession of a contractual clause for systemic capacity introduces severe operational risks (Government Digital Service, 2025; European Commission, 2025a; Office for Artificial Intelligence, 2020; OECD, 2015). Three distinct feasibility constraints follow: public authorities frequently lack the domestic engineering capacity to execute deep audits; commercial vendors routinely resist disclosure, liability exposure, and data-reuse restrictions; and cross-jurisdictional variances in procurement and administrative procedures demand highly calibrated, context-specific legal implementation over standardised templates.

Consequently, central technical support institutions are critical. In the UK, the Government Digital Service (Government Digital Service, 2025; Cabinet Office, 2025b) provides centralised digital engineering support for public sectors. Globally, the European AI Office (European Commission, 2025b; Regulation (EU) 2024/1689) facilitates unified AI Act enforcement and technical guidance. Rather than displacing an authority’s inherent public duties, these bodies make them operational by supplying necessary expertise, standardised contract templates, and interpretive discipline (European Commission, 2025b; Office for Artificial Intelligence, 2020; OECD, 2015; OECD, 2025a).

A secondary limitation is contractual over-saturation. If every public procurement imposes exhaustive compliance, continuous traceability, and granular reporting burdens, the market will disproportionately favour large commercial incumbents and deepen long-term vendor dependency. Imposing highly customised, legally dense contract specifications inevitably inflates transaction and compliance costs (Arrowsmith, 2018), introducing structural market friction. This regulatory overhead risks choking off supplier participation, creating a chilling effect where dominant firms refuse public tenders to protect proprietary architectures (Schooner, 2001). Most critically, rigid auditing frameworks disadvantage SMEs and open-source startups lacking the capital reserves and institutional capacity to navigate complex public purchasing barriers or extensive regulatory compliance overheads (Kalvet et al., 2011). The solution requires preserving the non-derogable core whilst scaling peripheral compliance duties to sectoral risk and supplier maturity.

Further methodological limitations must be acknowledged. Rather than a validated empirical architecture, these integrated mechanisms serve as a normative blueprint testing the conceptual feasibility of public law procurement design. This study has not evaluated live, unredacted public AIaaS agreements due to commercial confidentiality defaults. Furthermore, contractual configurations require sectoral elasticity; internal resource routing algorithms demand fewer controls than rights-determinative welfare, border, taxation, or policing systems. These limits reinforce the need for public templates treating administrative legality as a standard baseline rather than a customised luxury (European Commission, 2025b; Office for Artificial Intelligence, 2020; OECD, 2015). Whilst this text leverages official, commercial, and practise-based web sources to map contemporary AIaaS infrastructure terms, these materials serve as practical indicators of market practise rather than formal doctrinal authority, corroborated alongside established legal frameworks, case law, and peer-reviewed scholarship to illustrate why structural technical intervention is required before automated systems fragment traditional public oversight.

Standardisation becomes urgent where agentic systems modify the structural nature of outsourcing. Public procurement can no longer assume a static software asset operating within a stable administrative frame; it increasingly confronts adaptive, multi-agent systems that challenge traditional concepts of delegation and accountability. This M2M economy shifts AIaaS from outsourcing discrete classification tasks to externalising adaptive decisional capacity across a dynamic chain of autonomous agents that retrieve data and interpret policy in real time. This structural fluidity exerts severe pressure on administrative legality along two specific fronts.

The first pressure is distributed opacity and cascading illegality. In multi-agent environments, the underlying rationale for an action is distributed across agentic hand-offs, rendering each sub-agent’s logic contingent on external proprietary outputs. Procurement agreements must therefore mandate joint and several evidentiary responsibility from the lead system integrator, requiring them to document, preserve, and produce the continuous logic chain of the ecosystem during an adverse citizen challenge (Cobbe et al., 2023; Public Law Project, 2024).

The second pressure is the vendor’s functional mutation into a delegated administrative gatekeeper. Real-time biometric surveillance, border monitoring, or algorithmic sensor streams translate proprietary evaluations directly into executive actions. Where agentic swarms modify welfare triage or border screening rules in real time, authorities require immediate contractual rights to interrogate, restrain, and override outputs; otherwise, adaptive automation directly undermines non-delegation principles (Yeung, 2018; Hildebrandt, 2020). The administrative duty of candour must be technically hardened to ensure agentic autonomy never functions as a shield against public review.

## 6 Conclusion

AIaaS and composable agent systems represent a profound privatisation of administrative capacity, outsourcing not just service delivery, but the foundational classificatory and inferential logic through which public law is applied. This digital relocation facilitates a structural legality gap, displacing core public-law cheques directly into private contractual infrastructures (European Commission, 2023; Farrell et al., 2023; United Nations, 2011). Consequently, the procurement agreement must function as a mechanism for normative restoration, preserving the non-derogable baselines of good administration—reason-giving, non-discrimination, transparency, and traceability—via the Three-Layer Accountability Framework.

As administrative power migrates into distributed M2M environments, this framework shifts the operational focus of public delegation doctrine. It establishes that administrative cognition cannot be contractually alienated without causing systemic legal deficits, rendering continuous technical traceability an essential prerequisite for the survival of the rule of law within automated governance (European Commission, 2025a; Cabinet Office, 2025b; OECD, 2025b). Good administration will survive only if public sector procurement is treated as an act of digital constitutionalism rather than a standard commercial transaction. By framing the contract as a strict constitutional boundary, this study demonstrates that automated privatisation requires explicit technical verification architectures. The primary theoretical contribution of this framework is the rejection of *post hoc* judicial review as a sufficient standalone safeguard, establishing instead that the *ex ante* procurement contract serves as the primary jurisdictional site where the state-citizen relationship is either preserved or surrendered to private discretion.

## Glossary

AI: Artificial intelligence
AIaaS: AI-as-a-Service
APA: Administrative Procedure Act (US)
API: Application programming interface
ATRS: Algorithmic transparency recording standard
CCM: Correction cost model
CI/CD: Continuous integration/continuous deployment
CJEU: Court of Justice of the European Union
DevOps: Development and operations
EU: European Union
FOIA: Freedom of Information Act
GDPR: General Data Protection Regulation
IP: Intellectual property
JSON: JavaScript Object Notation
LLM: Large language model
M2M: Machine-to-Machine
SaaS: Software-as-a-Service
SMEs: Small and Medium-sized Enterprises
UK: United Kingdom
US: United States
UTL: Unilateral Termination for Legality
