# Supplementary material for: The contractual vacuum: AI procurement and the erosion of good administration
Source: Front Artif Intell. 2026 Sep 23;9:1916534. doi: 10.3389/frai.2026.1916534 (PMC13642710; doi:10.3389/frai.2026.1916534)
Supplement: Supplementary file 1 [file Data_Sheet_1.PDF]

Supplementary Material: Model Clause Blueprint

Clause 1: Transitive Audit and Forensic Access Right (Layer 1: Validation)

1.1 Right of Forensic Inspection. The Vendor hereby implements OpenTelemetry logging standards within the computational stack. This architecture exposes model weight distributions and input vector spaces to automated public auditing scripts while protecting proprietary trade code through sandboxed secure execution environments. This right extends down the algorithmic supply chain to all underlying models, sub-agents, orchestrators, and third-party Application Programming Interfaces (APIs) that materially contribute to any final administrative recommendation or output.

1.2 Scope of Material to be Disclosed. Upon written notice following a contested administrative decision, the Vendor must provide secure, containerised access within twenty-four (24) hours to:

(a) The exact historical snapshot of the model configuration, relevant parameters, threshold settings, orchestration logic, and decision-state metadata utilised at the timestamp of the decision;

(b) Full validation methodology and bias regression testing logs against legally curated statutory benchmarks; and

(c) Secure technical credentials, structural parameters, and access protocols necessary to reproduce the decisional environment for forensic verification.

1.3 Override of Commercial Secrecy. The Vendor explicitly agrees that assertions of proprietary trade secrets, commercial confidentiality, or intellectual property rights shall not be used to withhold, redact, or obstruct the delivery of information required by the Public Authority to discharge its statutory duties of candour, explanation, or judicial review.

Clause 2: Unilateral Termination for Legality (UTL) (Layer 2: Operational Liability)

2.1 UTL Trigger Events. The Public Authority reserves an express priority right to terminate this Agreement without commercial penalty or exit fee where a material systemic legality failure is identified, including where:

(a) The AIaaS environment produces outputs that exhibit disparate impact or statistically significant bias against any protected characteristics defined under domestic equality law;

(b) The system undergoes a silent modification, unapproved retraining cycle, or autonomous optimisation that alters the practical application of the statutory standard under which it was procured;

(c) The Vendor fails to produce decision-specific explanatory metadata within the statutory timeframe for a citizen's appeal; or

(d) The Vendor fails to disclose material corporate engagement in dual-use or foreign military applications that generates an unmanageable conflict of interest with public trust or national security.

2.2 Neutral Transition Obligation. Upon invocation of this UTL clause, the Vendor must immediately activate the Neutral Transition Protocol. The Vendor must securely export all public sector data, transaction logs, and metadata states in a zero-lock-in, human-readable format, maintaining service continuity until a controlled system replacement is completed.

Clause 3: Refined Correction Cost Model (CCM) Indemnity (Layer 3: Restitutive Repair)

3.1 Liquidated Administrative Damages. In the event of an unapproved logic shift, unauthorised deletion or corruption of decision-state records, loss of auditability, or algorithmic error that materially impairs the Public Authority’s ability to reconstruct, review, or lawfully correct affected administrative decisions, the Vendor shall be liable for the reasonable fiscal and operational costs of structural remediation. The parties acknowledge that the precise losses resulting from a public law integrity failure are difficult to estimate at the time of contracting and therefore agree that this Correction Cost Model (CCM) constitutes a genuine pre-estimate of recoverable remediation costs and a proportionate liquidated damages mechanism, not a penalty.

3.2 Fiscal Formula for Re-Adjudication. The Vendor shall reimburse the Public Authority for reasonable manual re-adjudication costs calculated using the following formula:

\begin{equation}

\text{Total Liability} = N \times (H \times R) + E

\end{equation}

[Plain-text alternative: Total Liability = N × (H × R) + E]

where N denotes the number of affected administrative decisions requiring manual re-adjudication, H denotes estimated caseworker hours per decision, R denotes the applicable hourly remediation rate, and E denotes reasonable external audit, notification, and system-reconstruction costs.
